# Fractionated alpha and mixed beam radiation promote stronger pro-inflammatory effects compared to acute exposure and trigger phagocytosis

**DOI:** 10.3389/fncel.2024.1440559

**Published:** 2024-12-09

**Authors:** Mostafa Karimi Roshan, Sergey Belikov, Melissa Ix, Nicoletta Protti, Claudia Balducci, Richard Dodel, J. Alexander Ross, Lovisa Lundholm

**Affiliations:** ^1^Department of Molecular Biosciences, Centre for Radiation Protection Research, The Wenner-Gren Institute, Stockholm University, Stockholm, Sweden; ^2^Therapy Research in Neurogeriatrics, Chair of Geriatric Medicine, University Duisburg-Essen, Essen, Germany; ^3^Department of Geriatric Medicine, Center for Translational and Behavioral Neuroscience, University Duisburg-Essen, Essen, Germany; ^4^Department of Physics, University of Pavia, Pavia, Italy; ^5^Pavia Unit, National Institute of Nuclear Physics INFN, Pavia, Italy; ^6^Department of Neuroscience, Istituto di Ricerche Farmacologiche Mario Negri IRCCS, Milan, Italy

**Keywords:** radiation, microglia, inflammation, DNA damage, cGAS-STING, phagocytosis

## Abstract

**Introduction and methods:**

Aiming to evaluate safety aspects of a recently proposed approach to target Alzheimer’s disease, we mimicked a complex boron neutron capture therapy field using a mixed beam consisting of high- and low-linear energy transfer (LET) radiation, ^241^Am alpha particles (α) and/or X-ray radiation respectively, in human microglial (HMC3) cells.

**Results:**

Acute exposure to 2 Gy X-rays induced the strongest response in the formation of γH2AX foci 30 min post irradiation, while α- and mixed beam-induced damage (α:X-ray = 3:1) sustained longer. Fractionation of the same total dose (0.4 Gy daily) induced a similar number of γH2AX foci as after acute radiation, however, α- or mixed irradiation caused a higher expression of DNA damage response genes CDKN1A and MDM2 24 h after the last fraction, as well as a stronger decrease in cell viability and clonogenic survival compared to acute exposure. Phosphorylation of STING, followed by phosphorylation of NF-κB subunit p65, was rapidly induced (1 or 3 h, respectively) after the last fraction by all radiation qualities. This led to IL-1β secretion into the medium, strongly elevated expression of pro-inflammatory cytokine genes and enhanced phagocytosis after fractionated exposure to α- and mixed beam-irradiation compared to their acute counterparts 24 h post-irradiation. Nevertheless, all inflammatory changes were returning to basal levels or below 10–14 days post irradiation.

**Discussion:**

In conclusion, we demonstrate strong transient pro-inflammatory induction by daily high-LET radiation in a microglia model, triggering phagocytosis which may aid in clearing amyloid beta, but importantly, from a safety perspective, without long-term alterations.

## Graphical abstract

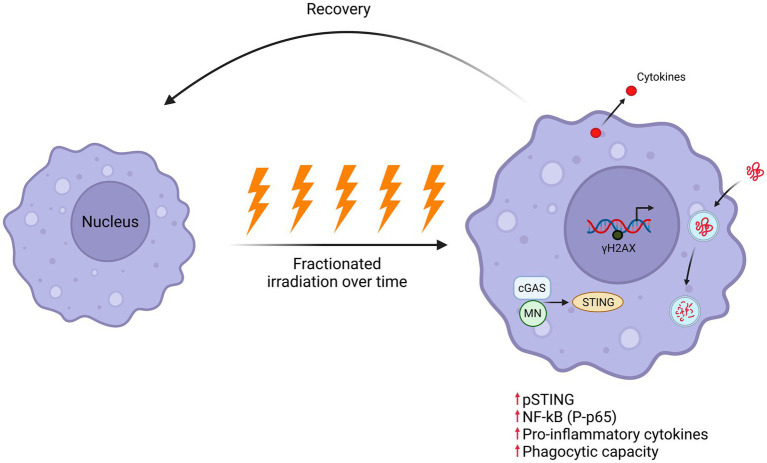

## 1 Introduction

Radiation therapy (RT) is one of the most common tools for cancer treatment, used either alone or in combination with surgery, hormone therapy, chemotherapy, and immunotherapy (Pereira et al., 2014). Technological advances of the last decades enable the delivery of RT to tumors with great precision; however, the dose tolerance of the healthy tissue remains an obstacle on the path to maximal eradication of tumor cells. An obvious approach is to use new modes of RT deposition as well as the employment of heavy particles. Low-LET radiation-induced double-strand breaks (DSBs) generally undergo rapid repair, primarily through the mechanism of non-homologous end joining (NHEJ), and are less toxic to cells (Lees-Miller and Meek, 2003). High-LET radiation focuses energy along particle trajectories, causing localized and clustered DNA damage and, thus, is more lethal than similar doses of low-LET radiation (Asaithamby and Chen, 2011). Phosphorylation of the histone variant H2AX at Ser-139, leading to the formation of γH2AX foci, represent an early cellular response to the induction of DSBs. DNA lesions also induce the activation of DNA damage response genes including *CDKN1A* (p21), *MDM2*, and *FDXR* (see Chen et al., 1994; O'Brien et al., 2018; Jebelli et al., 2022 for review).

One type of particle therapies currently in use for head and neck cancers and glioblastoma multiforme is boron neutron capture therapy (BNCT). BNCT principles were proposed as early as 1936, only 4 years after the discovery of neutrons. BNCT relies on ^10^B-containing compounds. It involves external irradiation with low energy (thermal) neutrons, resulting in the creation of a *de novo* complex beam containing high-and low-LET radiation (α-particles as well as lithium nuclei, and gamma rays, respectively) through reactions between neutrons and ^10^B in the target (tumor) cells (Malouff et al., 2021). In theory, exclusive boron delivery to target cells enables precise radiotherapy (RT) due to the sharp Bragg peak of high-LET particles with a range of about 10 microns. However, ^10^B uptake by normal tissues remains a challenge (Miyatake et al., 2016). In BNCT, the cumulative dose depends on the ^10^B concentration. To avoid adverse effects, the ^10^B concentration in tumors must be significantly higher than in healthy tissues (ratio ≥ 3). For the aims of the present study and for the experiments described later on, it is useful to note that ^10^B concentration in normal brain tissue during glioblastoma treatment typically ranges from 3.6 to 16.8 ppm (Elowitz et al., 1998; Bergenheim et al., 2005; Shimosegawa et al., 2016).

Brain tissue consists of various cells including neural cells, microglial cells, astrocytes, and oligodendrocytes, each with distinctive characteristics and functions. One and the same radiation quality is able to influence them differently and activate different mechanisms and pathways accordingly. In the adult brain, microglia, as innate immune cells in the central nervous system, contribute to the maintenance of homeostasis, immune surveillance, and regulation of neuroinflammation (Stupp et al., 2005). They play critical roles in brain health and disease, including cognitive processes. Microglia can undergo changes in response to various stimuli toward a range of phenotypes from anti-inflammatory to pro-inflammatory, which shape neuroinflammatory responses. Pro-inflammatory microglia promote neuroinflammation and neurotoxicity by releasing inflammatory cytokines and chemokines, whereas the anti-inflammatory microglia stimulate anti-inflammatory cytokines, healing, and neuroprotection (Paolicelli et al., 2022). To overcome the problem of restricted availability of primary human microglia cells, Tardieu lab established the human microglial clone 3 cell line (HMC3) by employing SV40 immortalization of human embryonic microglia cells. Since then, HMC3 cells have been comprehensively characterized and validated to present a relevant and robust model system for *in vitro* studies of brain cells, as the only commercially available immortalized human microglia cell model (Dello Russo et al., 2018).

Up to the present, BNCT has been almost exclusively used for the treatment of various forms of cancer. Recently, we proposed adapting NCT by ^10^B and ^157^Gd for Alzheimer’s disease (AD), the most common cause of dementia, within the EU-funded NEutron Capture-enhanced Treatment of neurotoxic Amyloid aggRegates (NECTAR) project. Existing AD treatment options are primarily symptomatic and provide only moderate benefits. We propose to employ the synergy between an external beam of low-energy neutrons with ^10^B and ^157^ Gd-bearing compounds as amyloid beta (Aβ)-targeting agents, which allow for a boost in the radiation dose and switch the main quality of irradiation to high LET, specifically in Aβ-sites. We anticipate that this strategy could provide a bimodal treatment of the disease. Firstly, by local depolymerization of Aβ aggregates by high-LET particles, secondly, through a long-distance stimulation of the brain immune cells (microglia cells) by penetrating photons resulting from neutrons (Kim et al., 2020a; Kim et al., 2020b) and/or radiation from the high-LET particles. We reasoned that for a hypothetical treatment against AD, the ^10^B concentration in the brain tissue adjacent to the amyloid plaques should not exceed levels typical for normal brain tissue during glioblastoma treatment. Based on this and using a precautionary approach, a ^10^B concentration equal to 14 ppm was chosen. At this concentration, the characteristics of the TRIGA reactor in Pavia, Italy (used for the NECTAR project), pre-determine the proportion of high-LET radiation of the BNCT beam as 75%, thus, motivating the use of the high/low LET in the ratio of 3:1 (Bortolussi et al., 2018). In this study, we reconstruct the complex BNCT beam to elucidate the effects of high and low-LET radiation alone or in combination, delivered via single or fractionated radiation treatment protocols to human microglial HMC3 cells in order to understand the distinctive responses to different radiation qualities and schemes in terms of toxicity and induction of inflammatory response.

## 2 Materials and methods

### 2.1 Cell culture

The human microglial clone 3 (HMC3) cell line was purchased from ATCC (CRL-3304™), cultured in T75 flasks in Dulbecco’s Modified Eagle Medium (DMEM) (Sigma-Aldrich, Germany) supplemented with 10% defined bovine serum (Sigma-Aldrich, Germany) and 1% penicillin–streptomycin (Sigma-Aldrich, Germany) and subcultured every 3 days. The cells were maintained at 37°C and 5% CO_2_ and kept up to passage 20. Forty eight hours prior to irradiation cells were seeded on glass coverslips (ORSAtec, Germany) at the following densities (per well/coverslip): 2.0 × 10^5^ cells for the γH2AX and phagocytosis assay, 2.5 × 10^5^ cells for acute irradiation, and 1.0 × 10^5^ for fractionated irradiation. To avoid cell overgrowth for fractionated irradiation, the cells were always subcultured 3 h after the third irradiation; and after the last fraction, the cells were replated in 6-well plates and 96-well plates for clonogenic and resazurin assay, respectively. A part of the cells was collected for gene expression, protein expression analysis and/or flow cytometry analysis 1, 3, 6, or 24 h after irradiation and kept at −80°C. The supernatant after cell pelleting was collected 24 h post-irradiation and kept at −80°C for ELISA assay. Part of the irradiated cells was maintained for further analysis at later time points (10 and 14 days), then harvested and kept at −80°C.

### 2.2 Irradiation protocols and sources

Coverslips with growing cells were placed on a polyamide disk subsequently covered with a 2.5-um thick Mylar foil and placed in direct contact with the α-particle source using a motor device (see Staaf et al., 2012 for details). An Am-241 source was utilized for the α-particle irradiation, with a dose rate of 0.223 Gy/min and an average LET of 91 keV/μm. X-ray irradiation was performed using an X-ray tube which was operated at 190 kV, 4.0 mA without the inbuilt aluminum filter and with the dose rates of 0.068 Gy/min and 0.052 Gy/min at the bottom-and top-shelf position, respectively. The mixed beam irradiation was performed by using both of the sources simultaneously. The X-ray tube was always switched on when the disk with coverslips was at the top position contacting the α-source (Staaf et al., 2012).

### 2.3 Cell viability and cell survival assay

Three hours post-irradiation the cells were harvested from the coverslips by trypsinization and reseeded in triplicate at the concentrations of 600, 1,200, and 2,400 cells per well in a 96-well flat bottom plate and left in the incubator for 5 days. Resazurin reagent was added at a final concentration of 0.1 mg/mL and the plate was incubated for 4 h at 37°C in the dark. Subsequently, plates were analyzed using a microplate reader (BMG Labtech, Germany) to measure fluorescence, resazurin has ex/em of 530–560/590 nm.

In parallel, HMC3 cells were replated in duplicate at a density of 200, 400, and 800 cells per well for non-irradiated and 800, 1,600, and 3,200 cells/well for irradiated cells using the fractionated scheme, and 200, 400, and 800 cells/well for single-dose exposure scheme. Colonies were allowed to form for 10 days, then fixed with 1:3 acetic acid-methanol fixative and subsequently stained with 5% Giemsa in 25% methanol for 30 min. The colonies were counted using the ImageJ macro countPHICS (Brzozowska et al., 2019).

### 2.4 γH2AX assay

Cells were seeded on square 22 mm glass coverslips 48 h prior to irradiation and fixed 10 min, 30 min, and/or 24 h after exposure in 70% EtOH. Treatment with 0.2% Triton X-100, 5 min was used for permeabilization of the cells on the coverslips. Subsequently, the cells were rinsed with PBS and stained with anti-phospho-Histone H2AX antibody Ser139 (16-202A, Sigma-Aldrich, Germany, 1:200) in 2% BSA in PBS at 37°C for 30 min followed by rinsing with PBS. Samples were incubated with secondary anti-mouse IgG fluorescein isothiocyanate (FITC) (Sigma-Aldrich, Germany, 1:800) in 2% BSA in PBS. Coverslips were counterstained with DAPI and mounted on an objective glass using VECTASHIELD^®^ containing DAPI (Vector Laboratories, United States). Images were captured using a fluorescent microscope using a 100X objective (Nikon Eclipse E800; Nikon, Tokyo, Japan), and the total number of γH2AX foci per cell was scored using a macro for ImageJ version 1.43u, as previously described (Sollazzo et al., 2017). From each group, at least 50 cells were randomly selected for analysis, ensuring an equal number of cells per group.

### 2.5 Gene expression analysis

RNA extraction was performed using the E.Z.N.A. Total RNA Kit I (Omega Bio-Tek, United States). cDNA was synthesized using a High-Capacity cDNA Reverse Transcription Kit (Thermo Fisher Scientific, United States). The reaction mix consisted of primers, cDNA, and 5x HOT FIREPol^®^ EvaGreen^®^ qPCR Supermix (Solis BioDyne, Estonia). Real-time PCR was carried out in 96-multiwell plates in duplicate using a LightCycler^®^ 480, and the temperature protocol was starting at 95°C for 15 min, followed by 40 cycles of 95°C for 15 s, 60°C for 20 s, and 72°C for 20 s. Primers were toward *CDKN1A*, *MDM2*, *FDXR*, *IL-18*, *IL-12α*, *IL-10*, *IL-1β*, *CD163*, and *CD206* (see Sollazzo et al., 2017; Panda et al., 2021; López-Riego et al., 2023; Roshan et al., 2023 for primer sequences) (LGC Biosearch Technologies, Denmark). The data was normalized against *18S* rRNA and *GAPDH*. The 2^−∆∆Ct^ method was used to calculate the relative fold gene expression (Livak and Schmittgen, 2001).

### 2.6 ELISA assay

Analysis of secretion of IL-1β protein in the media was carried out using a commercially available IL-1β ELISA kit (Sigma-Aldrich, Germany) based on an antibody sandwich method using microtiter plates, coated with the IL-1β cytokine. Supernatants from samples were concentrated using a vacuum centrifuge with the aim of concentrating IL-1β in samples, frozen samples (−80°C) were directly placed in the vacuum centrifuge and centrifuged for 3 h, in order to reduce the volume from 1,000 to 200 μL. Each sample was assayed in duplicate, according to the manufacturer’s instructions. Absorbance was measured by a microplate reader (BMG Labtech, Germany) at 450 nm.

### 2.7 Western blot

Irradiated and non-irradiated cells were trypsinized, harvested, and lysed directly with loading buffer (10% SDS, 500 mM DTT, 50% glycerol, 500 mM Tris–HCL, and 0.5% bromophenol blue dye). Protein separation was achieved using 4–12% Bis-tris gradient gels in 1xMES running buffer (Invitrogen^™^, United States). The separated proteins were then transferred to a nitrocellulose membrane (Thermo Scientific, United States). Subsequently, the membrane was blocked using Odyssey^®^ blocking buffer (Odyssey Blocking Buffer from LI-COR, UK) and Tris-buffered saline containing 0.05% Tween (TBST) in a 1:1 ratio at room temperature for 1 h.

Probing was conducted using the following primary antibodies overnight at 4°C: Phospho-STING (Ser366, Thermo Fisher Scientific, 1:500), p65 (Sigma-Aldrich, 1:500) and P-p65 (Sigma-Aldrich, 1:500) as well as GAPDH (G8795, Sigma-Aldrich, 1:20,000). Subsequent to the primary antibody incubation, probing with secondary antibodies, infrared dye-conjugated goat anti-rabbit or donkey anti-mouse secondary antibodies (LI-COR, Cambridge, UK, 1:15,000) was carried out for 1 h at room temperature. The membranes were scanned, and the levels of proteins were analyzed using the Odyssey^®^ S Infrared Imaging System (LI-COR) and quantified with Image Studio™ Lite version 5.2 (LI-COR, UK).

### 2.8 Phagocytosis assay

Phagocytosis assay was conducted using a commercial phagocytosis assay kit (Sigma-Aldrich, Germany). Zymosan particles labeled with a red fluorophore, enabling detection and measurement through a fluorescent microscope, spectrophotometer, or flow cytometry, were used. Seventy two hours after the last fraction of irradiation, HMC3 cells were harvested and plated in a black 96-well plate with a clear bottom for spectrophotometry, and on square coverslips for fluorescent microscopy, suspended cells were removed after an hour of incubation. Subsequently, cells were treated with 5 μL of Zymosan and incubated overnight. The wells were rinsed with the provided phagocytosis buffer according to the manufacturer’s instruction. Quantification was performed in duplicate using a microplate reader (SpecraMax i3x, United States) at an ex/em wavelength of 540/570 from the bottom. In addition, the coverslips were immunostained with Anti-α-Tubulin−FITC (F2168, Sigma-Aldrich, Germany), counterstained with DAPI, and mounted on the objective glass slides for fluorescent microscopy.

### 2.9 Flow cytometry

HMC3 cells were analyzed on a CytoFLEX flow cytometer (Beckman Coulter, Germany) using the CytExpert software. Cells in anti-inflammatory state were defined by CD206 (Alexa Fluor® 700 anti-human CD206 (MMR) Antibody, BioLegend, Koblenz) surface marker. Intracellular staining for TNF-α (APC anti-human TNF-α Antibody), IL-1β (FITC anti-human IL-1β Antibody) and IL-10 (PE/Dazzle^™^ 594 anti-human IL-10 Antibody, all from BioLegend) was performed after treatment with Fixation/permeabilization Kit (BD Biosciences) according to the manufacturer’s manual.

### 2.10 Statistical analysis

Data normality distributions were confirmed using the Shapiro–Wilk test. Therefore, statistical analysis was carried out using two-way ANOVA and one-way ANOVA considering the number of variables, and multiple comparisons were corrected by Bonferroni’s and Tukey’s tests, respectively (GraphPad prism ver. 10.1.0). *p* value < 0.05 was considered statistically significant when comparing α or mixed beams to X-rays (^*^), or these groups to themselves at the different time points. Comparisons to control (^#^) were performed as indicated in the figure legends. Cell viability data points for X-ray and α-particles and also the survival curve of X-ray irradiated cells were fitted to a linear quadratic equation $S=e^{-\left(\mathrm{aD}+\beta D^{2}\right)}$ where D is the total radiation dose in Gy, α and β are fitting coefficients. The survival curve of α-irradiated cells and also residual γH2AX foci data points were fit to a linear equation $S=e^{-\left(\alpha D\right)}$, α is a fitting coefficient.

**Figure 1:**
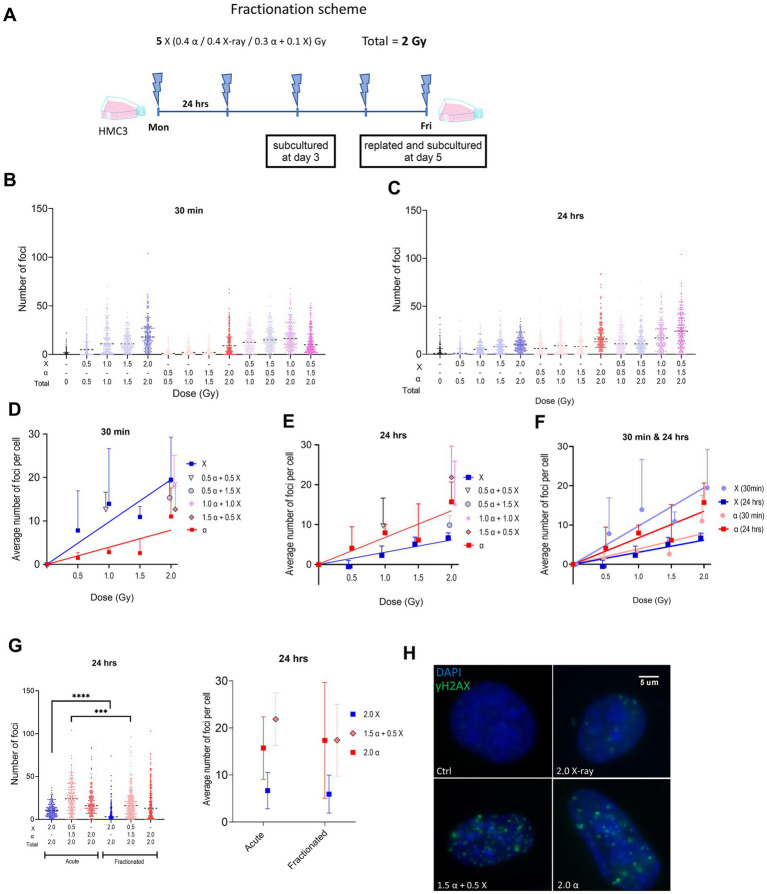
**(A)** Schematic representation of the fractionated irradiation protocol. **(B,C)** Violin plots showing γH2AX foci numbers per cell after different irradiation modalities using all values from three experiments (50 cells per experiment) at 30 min **(B)** and 24 h **(C)** after acute irradiation of HMC3 cells. Dashed lines in violin plots represent median. **(D,E)** Plots illustrate the same data using mean ± SD for three independent biological experiments, after subtraction of control foci. Lines represent fitted lines for X-ray and alpha particle data points using linear regression. **(F)** Plot displays both dose–response curves of X-ray and alpha particles at different time points. **(G)** γH2AX foci formation at 24 h after the last fraction using a total dose of 2 Gy given by an acute or fractionated irradiation protocol displayed as a violin plot (as in **B,C**) or mean ± SD (as in **D,E**). **(H)** Representation of fluorescence microscopy of the nucleus of HMC3 after fractionated irradiation with different radiation qualities. Symbols are nudged for transparency. Asterisks represent significance at the levels of *** < 0.001 and **** < 0.0001. Statistical analysis was carried out using two-way ANOVA and multiple comparisons were corrected by Bonferroni’s test.

**Figure 2:**
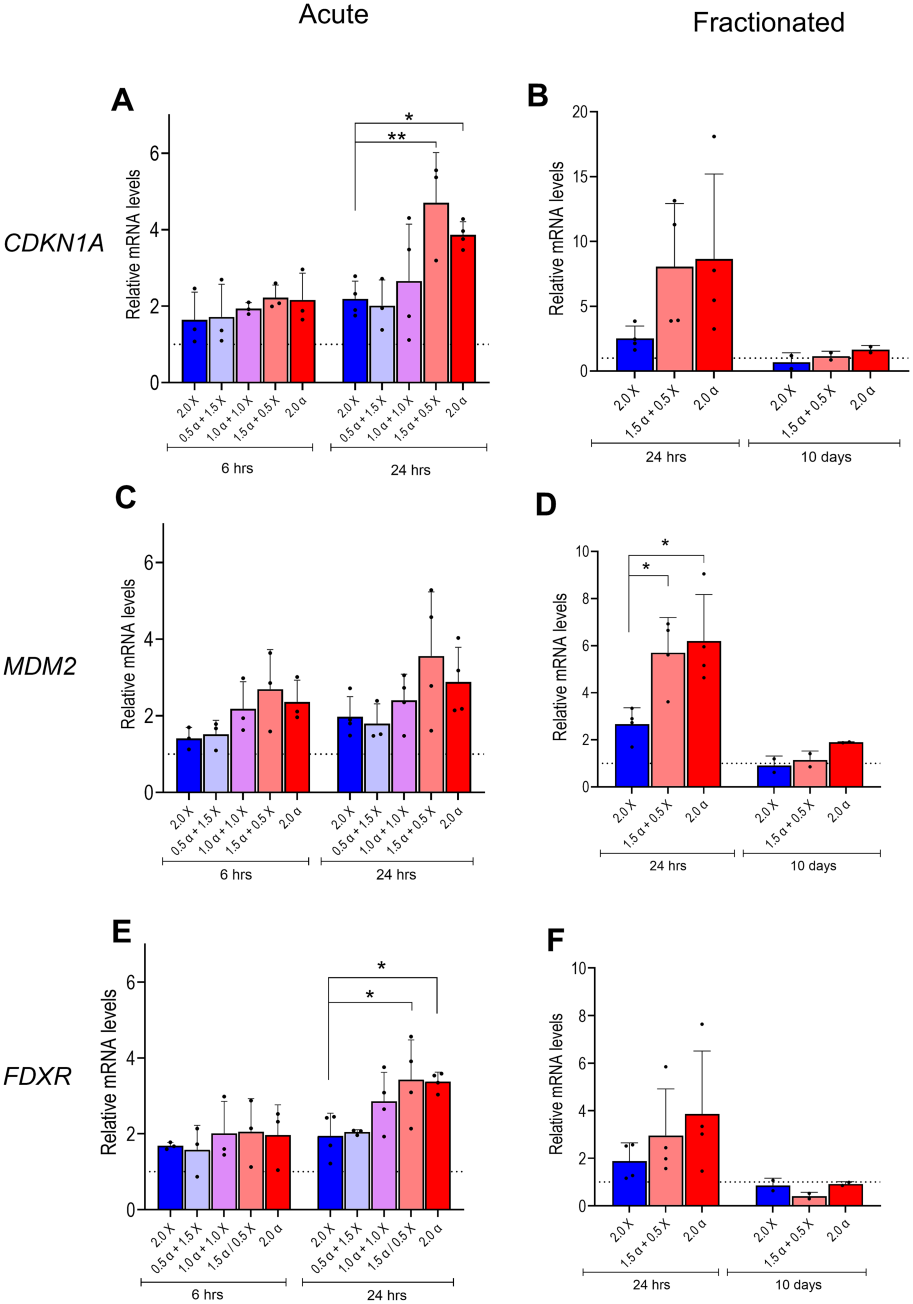
mRNA expression of DNA damage/repair genes. Relative levels of CDKN1A, MDM2, and FDXR mRNA in HMC3 cells were assayed 6 and 24 h after acute exposure to radiation beams comprising different proportions of α-particles and X-rays as indicated **(A,C,E)**, or 24 h and 10 days after fractionated irradiation **(B,D,F)**. Bars represent mean results and symbols represent individual values from independent experiments (*n* = 3–4). Error bars signify SD. Asterisks represent significance at the levels of * < 0.05 and ** < 0.01. Statistical analysis was carried out using two-way ANOVA and multiple comparisons were corrected by Bonferroni’s test.

**Figure 3:**
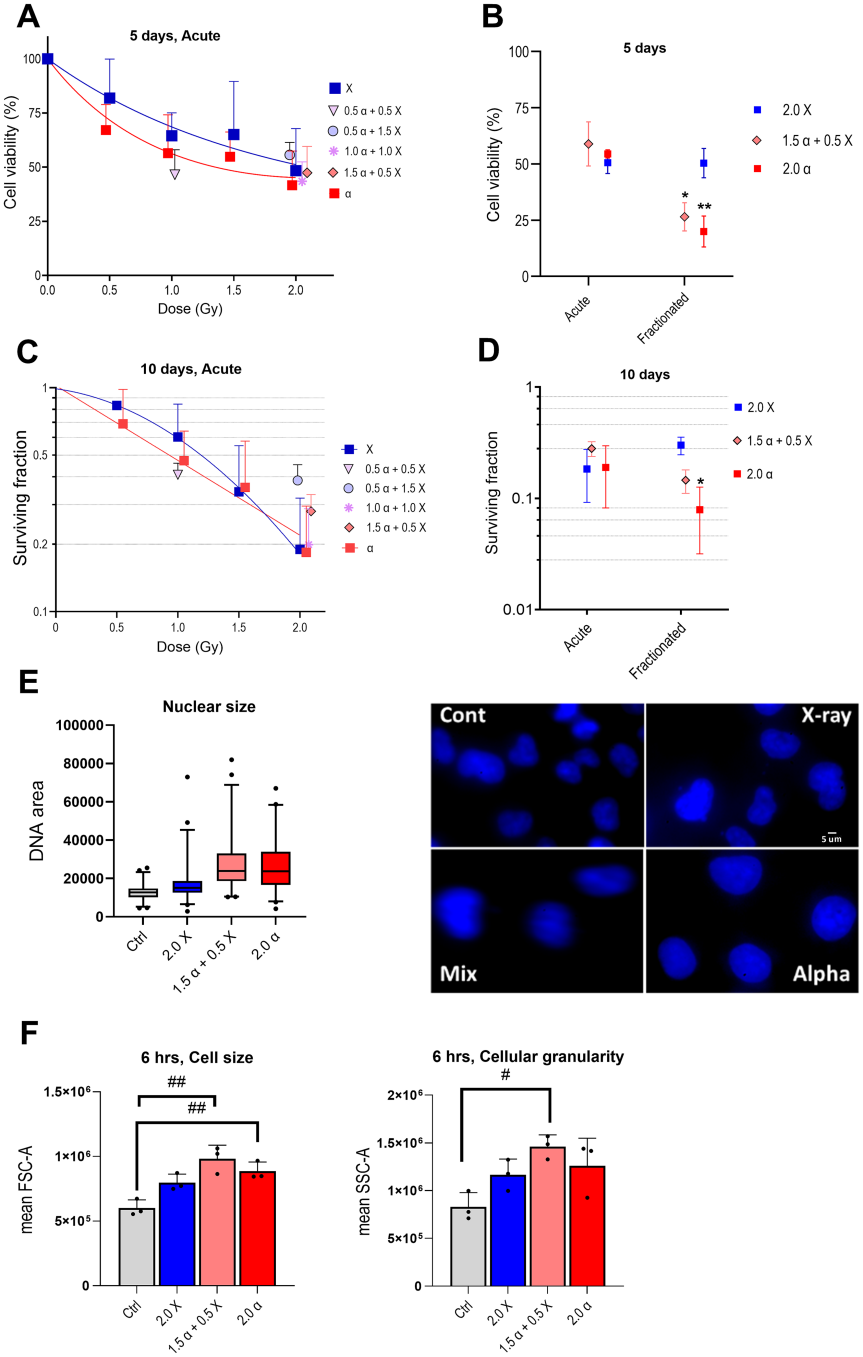
Radiation reduces cell viability and clonogenic survival and enlarges the nuclear and cell size of HMC3 cells. Cell viability plots are depicted after acute **(A)** or acute versus fractionated **(B)** exposures, and clonogenic survival after acute **(C)** or acute versus fractionated **(D)** exposures. **(E)** Nuclear size box plot and the representative fluorescent microscopy of DAPI stained nucleus of HMC3 cells (100X objective). **(F)** Cell size (FSC-A; forward scatter area) and cellular granularity/complexity (SSC-A; side scatter area) of α, X-ray, and mixed beam-irradiated HMC3 cells 6 h after the fractionated scheme (*n* = 3). Error bars signify SD. Asterisks represent significance for alpha particle and mixed beam compared to X-ray at the levels of * < 0.05 and ** < 0.01 and hashtags shows significance between non-irradiated and irradiated groups at the levels of # < 0.05 and ## < 0.01. Statistical analysis was carried out using two-way ANOVA and multiple comparisons were corrected by Bonferroni’s test for cell viability and survival. One-way ANOVA was applied to analyze cell size and granularity.

**Figure 4:**
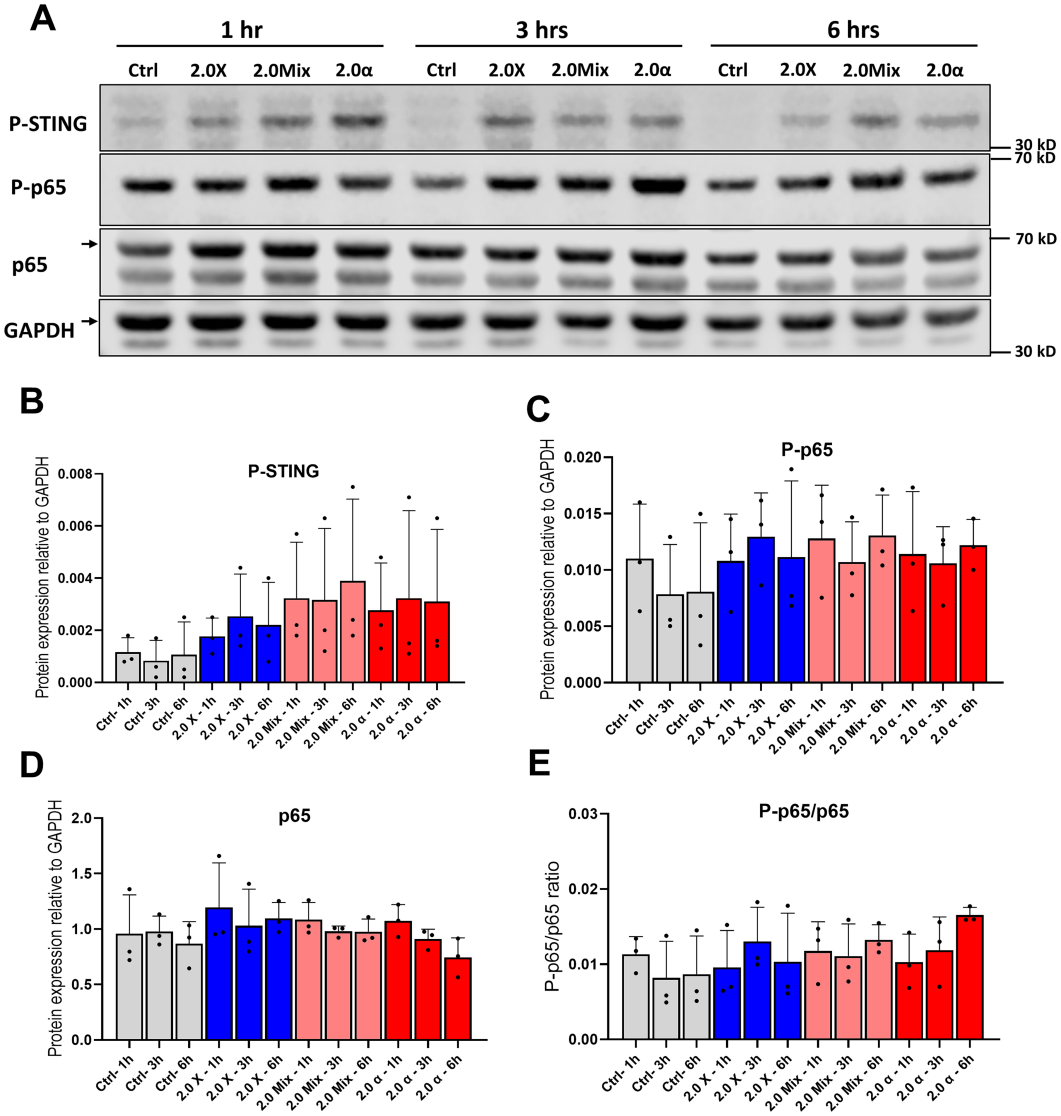
Protein expression and phosphorylation of p65 subunit of NF-κB and STING. **(A)** Representative western blot of p65 and STING at 1 h, 3 h, and 6 h after exposure to the total 2 Gy. **(B)** Densitometric quantification of relative changes in phosphorylation of STING and **(C,D)** protein expression and phosphorylation of p65. **(E)** The ratio of phosphorylation to expression of p65. Symbols represent mean results from three independent experiments. Bars represent mean results and symbols represent individual values. Error bars signify SD.

## 3 Results

### 3.1 Experimental design

To mimic the effect of BNCT in healthy tissue we used a high-LET/low-LET, i.e., α/X-rays ratio equal to three based on the abovementioned consideration, and most of the experiments were conducted using this ratio. Two irradiation protocols were employed: acute (single dose) and fractionated as shown in Figure 1A. Mixed beam irradiations were performed by simultaneous use of both high-and low-LET sources. Routinely, a 2 Gy dose was delivered to cells either in “one shot” (acute protocol) or in five consecutive daily fractions of 0.4 Gy (fractionated protocol).

**Figure 5:**
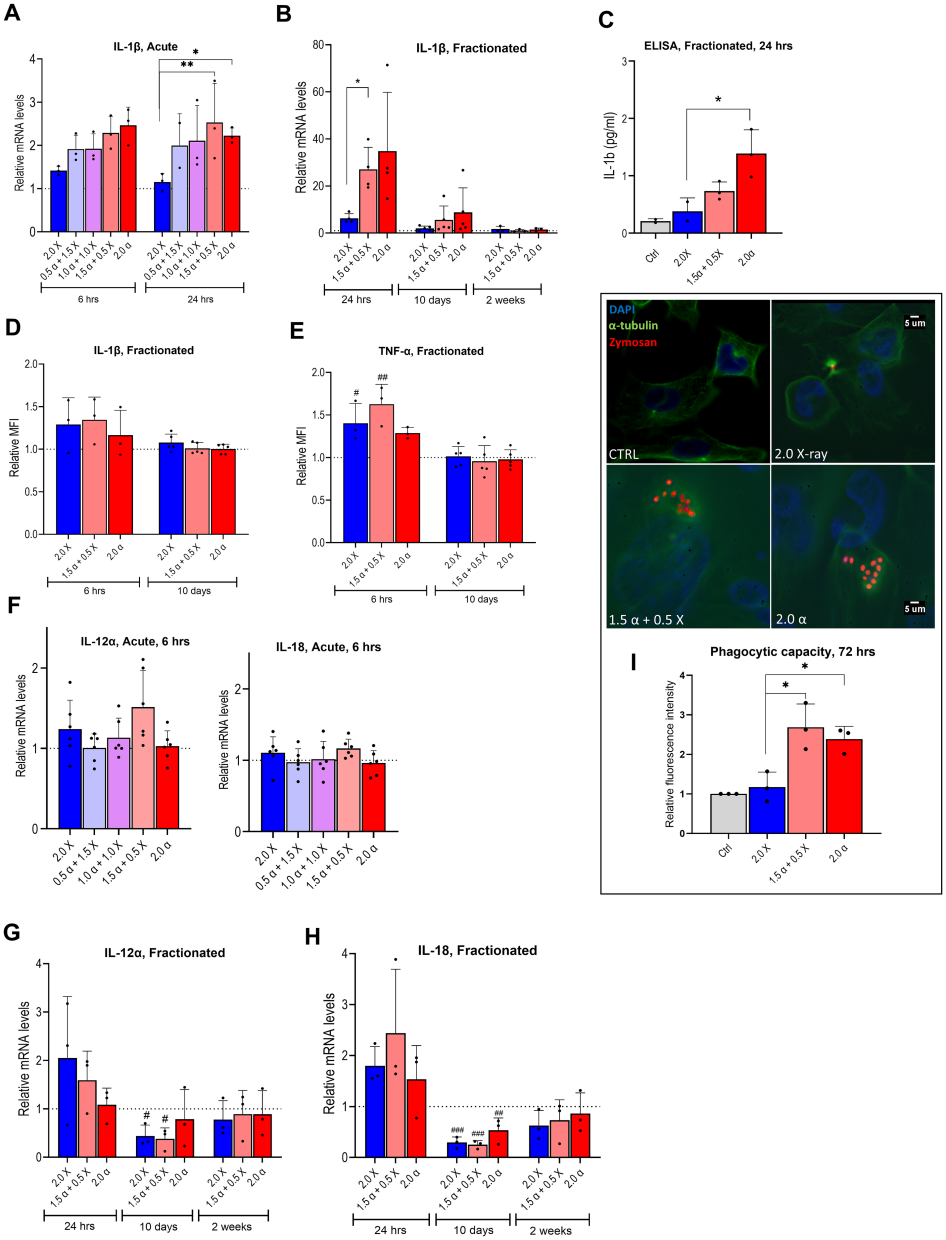
mRNA expression of pro-inflammatory markers and phagocytic ability of irradiated HMC3 cells. Relative mRNA levels of IL-1β in HMC3 cells were assayed **(A)** 6 h and 24 h after acute exposure to radiation beams with different proportions of α-particles and X-rays as indicated or **(B)** 24 h, 10 days and 14 days after fractionated irradiation. **(C)** HMC3 cell culture supernatants were collected after fractionated irradiation with indicated doses of irradiation and assayed for IL-1β secretion by ELISA (*n* = 3). Intracellular protein levels of IL-1β **(D)** and TNF-α **(E)** at 6 h and 10 days after fractionated irradiation, assayed by flow cytometry and presented as relative median fluorescence intensity (MFI) (*n* = 3–5). **(F–H)** Relative mRNA levels of IL-12α and IL-18 in HMC3 cells were assayed, 6 h **(F)** after acute exposure to radiation containing different proportions of α-particles and X-rays as indicated or 24 h, 10 days, and 14 days **(G,H)** after fractionated irradiation (*n* = 3–6). **(I)** Fluorescence spectroscopy results which are presented as relative fluorescence intensity indicate the phagocytic capacity of the HMC3 cells 72 h after the fractionated irradiation, fluorescence microscopy images show the α-tubulin (green), nucleus (blue), and Zymosan (red) using a 60X objective for fluorescence microscopy (*n* = 3). Bars represent the means and symbols represent individual values from independent experiments. Error bars signify SD. Asterisks represent statistical significance at the level of * < 0.05 and ** < 0.01 in the indicated comparisons and hashtags show statistical significance between the groups and the control # < 0.05, ## < 0.01, ### < 0.001. Statistical analysis was carried out using one-way ANOVA and multiple comparisons were corrected by Tukey’s test for phagocytosis and ELISA and two-way ANOVA for mRNA expression levels.

### 3.2 DNA damage and γH2AX foci formation

We first characterized the formation of double strand breaks (DSBs) in acutely irradiated HMC3 cells by analysis of γH2AX foci. Cells were irradiated with either α-particles or X-rays alone (0.5 to 2.0 Gy) or using a mixed beam with different proportions of low-and high-LET components, namely 25/50/75% high LET with a total dose of 2 Gy, as well as 50% of each with a total dose of 1 Gy. Foci were quantified at 30 min and 24 h post irradiation. The results of γH2AX quantification are shown in Figures 1B–F. At all radiation modalities and exposure levels we observed a significant increase in the number of foci compared to non-irradiated samples. A clear tendency toward a higher number of foci was observed in cells 30 min after irradiation with X-rays compared to α-particles. The numbers of foci detected in cells irradiated with the mixed beam at different α:X-ray ratios were comparable but slightly higher than the numbers we obtained for samples that only received the dose of the X-ray component in a mixed beam. At 24 h after irradiation the number of foci for X-ray irradiated cells decreased, whereas the number of α-particle induced ones tended to increase as indicated in Figures 1C,E,H. We concluded that the higher the proportion of α-particles in the mixed beam, the higher levels of γH2AX foci remained after 24 h.

We also compared the DSB formation when using acute versus fractionated protocols. HMC3 cells received a total of 2 Gy of α-, X-ray, or mixed beam (α: X-ray = 3:1) radiation as a single dose or in five equal consecutive fractions. Mean foci numbers detected 24 h post irradiation by both protocols were very similar as shown in Figure 1G. However, this mean for the mixed beam is slightly lower in the fractionated setup. Interestingly, analysis of the overall population using a violin plot reveals a statistically significant reduction when using fractionation for both X-ray and mixed beam.

### 3.3 Expression of DNA damage response genes

The expression of three genes known to be among the first responders to radiation-induced DNA damage was assessed. At 6 h post acute irradiation, we observed a noticeable upregulation of all tested genes. The overall expression pattern indicated a trend toward elevated gene expression in samples that received doses with high proportions of α irradiation (1.5 α + 0.5 X-ray and 2.0 α) as shown in Figures 2A,C,E. The tendency became even more pronounced 24 h post irradiation; the difference in RNA expression between samples irradiated with low-LET radiation (2.0 X-ray) and samples that received high proportions of α-radiation reached statistical significance for the CDKN1A and FDXR genes (Figures 2A,E). We also assessed the mRNA levels of the selected DNA damage response genes in cells irradiated via the fractionation scheme 24 h and 10 days after the last irradiation. Similar to acute exposure, X-ray irradiated cells showed a moderate increase of mRNA levels (2–2.5-fold) 24 h post irradiation for all tested genes, while at 10 days post irradiation, expression levels returned to basal levels as indicated in Figures 2B,D,F. In cells that received doses with high proportions of α-radiation (1.5 α + 0.5 X-ray and 2.0 α), gene expression was even more upregulated 24 h post irradiation and decreased to basal levels 10 days after the delivery of the last fraction. However, statistical difference between X-ray irradiated cells and those exposed to high proportions of α-irradiation was observed only for the MDM2 gene. Interestingly, the average fold changes in CDKN1A and MDM2 gene expression in response to mixed or α-irradiation approximately doubled at 24 h after fractionated versus acute exposure.

**Figure 6:**
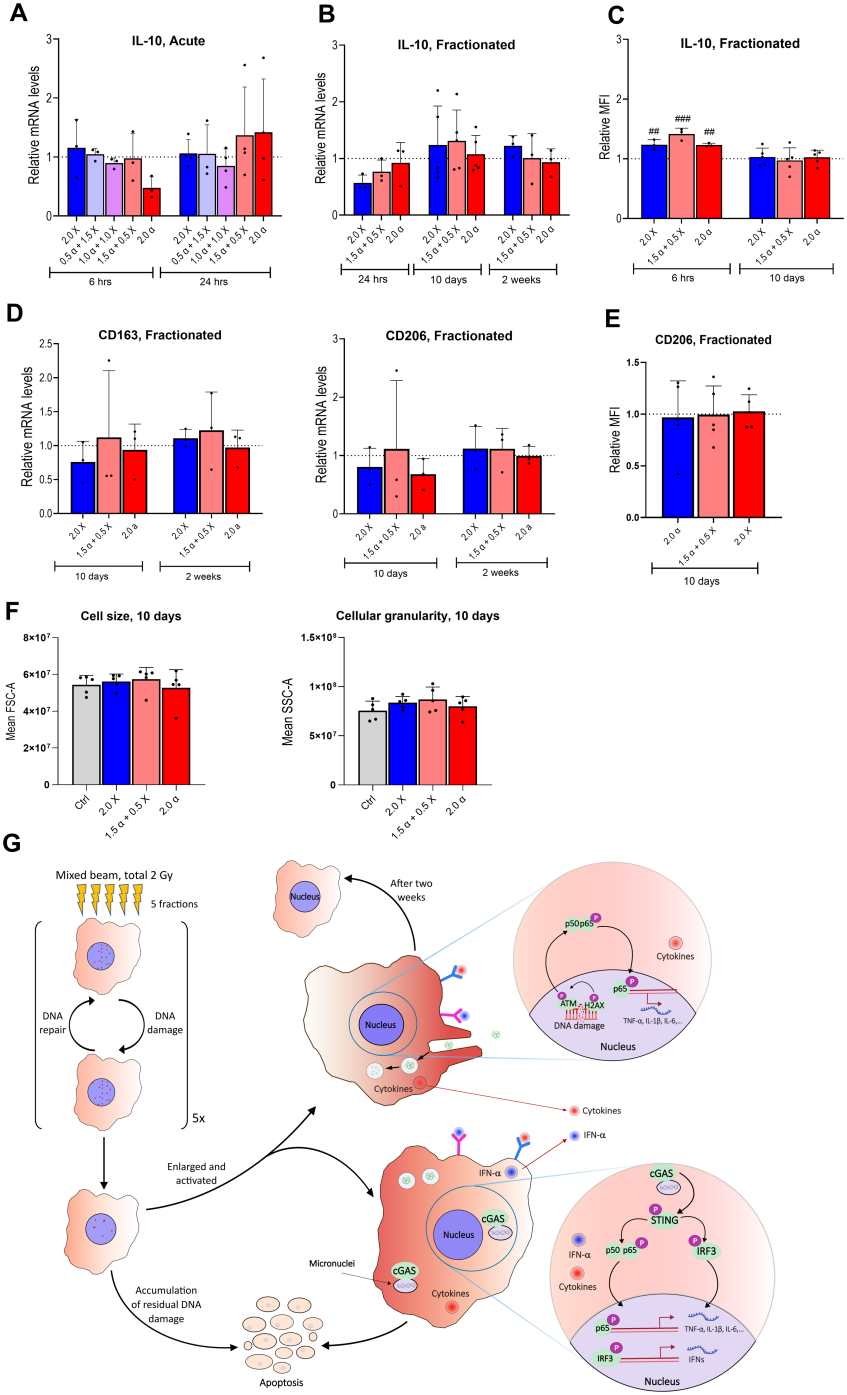
Few significant differences in mRNA expression levels of anti-inflammatory markers after either acute or fractionated irradiation. **(A)** Relative mRNA levels of IL-10 at 6 and 24 h post acute exposure to α-particles and X-rays alone and in combination with the total dose of 2 Gy. **(B)** mRNA levels of IL-10 shown at three different time points from 24 h to 14 days post fractionated irradiation (*n* = 3–6). **(C)** Intracellular levels of IL-10 protein after fractionated irradiation at 10 and 14 days after fractionated irradiation, assayed by flow cytometry and presented as relative median fluorescence intensity (MFI) (*n* = 3–5). **(D)** Relative mRNA levels of CD163 and CD206 at 10 and 14 days after fractionated exposure. **(E)** CD206 at 10 days after fractionated exposure, assayed by flow cytometry and presented as relative MFI (*n* = 5). **(F)** Cell size (FSC-A; forward scatter area) and cellular granularity/complexity (SSC-A; side scatter area) of unirradiated, α, X-ray and mixed beam-irradiated HMC3 cells at 6 h after the fractionated exposure. Bars represent mean results and symbols represent individual values. Error bars signify SD. Hashtags show statistical significance between the groups and the control ## < 0.01, ### < 0.001. Statistical analysis was carried out using two-way ANOVA and multiple comparisons were corrected by Bonferroni’s test. **(G)** Summarizing figure describing the DNA damage and inflammatory response up to 2 weeks after fractionated high-LET radiation exposure.

### 3.4 Radiation reduces cell viability and colony-forming ability of HMC3 cells and alters the cell and nuclear size

Firstly, HMC3 cells were exposed to increasing single doses of α- and X-ray radiation (acute protocol) to assess cell viability. Cell viability gradually decreased with the increase of X-ray radiation dose compared to the non-treated control cells. The decrease was somewhat more pronounced in cells exposed to α-particles. Clonogenic survival experiments showed the same tendency. As anticipated for high-LET irradiations, experimental data from α-irradiated samples could be fitted with a linear model. However, as shown in Figure 3A, the observed difference between two irradiation modalities was not as dramatic as could be expected based on previous studies (Goodhead, 1999; Roobol et al., 2020). Interestingly, our data from HMC3 cells suggests a relative biological effectiveness (RBE) of α-radiation to X-rays for 20% survival being around one (see Figure 3C). This indicates that high-LET α-particle radiation is not more effective than low-LET X-rays in this experimental context, at least at the highest tested dose.

Next, we compared the effectiveness of the fractionated irradiation protocol against the acute one. Cells were exposed to 2 Gy of α-, X-ray, or mixed beam (total 2 Gy; α:X-ray = 3:1) radiation, and the results are presented in Figures 3B,D. Both experimental approaches argue for minor differences in the cell viability and clonogenic survival following X-ray irradiation, only a tendency toward a sparing effect was evident from clonogenic survival. Contrary to this observation, fractionated irradiation with α- and mixed beam radiation (75% of α-particles) resulted in a prominent decrease in both cell viability and clonogenic survival compared to acute exposure. These findings appeared somewhat contradictory to γH2AX foci results (see Figure 1G) which demonstrated that mean foci numbers, i. e. the levels of DNA damage detected 24 h after irradiation via either of the protocols were relatively similar, yet the long-term effects at the gene expression and cell viability/survival level clearly differed. Interestingly, we noticed a change in the nuclear and cell size of HMC3 cells after fractionated radiation exposure, particularly more pronounced after high-LET compared to low-LET radiation, as depicted in Figures 3E,F. Additionally, both cell and nuclear sizes exhibited a similar trend, with the most notable increase observed after mixed beam radiation. An increase in cell size has previously been reported as a feature of activated microglia (Davis et al., 2017). Furthermore, our microscopic observations showed an increased number of microglial cells with amoeboid morphology in the alpha and mixed beam irradiated groups (data not shown), which aligns with elevated levels of pro-inflammatory cytokines and enhanced phagocytosis. However, confirming microglial activation requires further investigation using specific markers. Activation of cGAS-STING and NF-κB pathways in irradiated HMC3 cells.

To investigate the cyclic GMP-AMP synthase (cGAS)—stimulator of interferon genes (STING) and NF-κB pathway activation in HMC3 cells after exposure to fractionated irradiation and the kinetics of the activation, phosphorylation of STING, as well as phosphorylation and protein expression of the p65 subunit of NF-κB were analyzed at 1, 3 and 6 h after the last fraction of irradiation (Figure 4A). Our results regarding phosphorylation of STING indicated that the activation of STING occurred earlier and also at a higher magnitude compared to the NF-κB pathway (Figure 4B). Furthermore, the phosphorylation of p65 showed a slight increase time independently compared to non-irradiated cells (Figures 4C,E). Only minor changes were seen in the total protein expression of p65 (Figure 4D). Moreover, the ratio of P-p65/p65 after exposure to mixed beam and α-irradiation appear to peak later in comparison with low-LET, analogous to the delayed DNA damage response. Full blots are shown in Supplementary Figure S1. It is important to note that, although trends and minor alterations in the protein expression and activation of NF-κB and STING were observed, these changes did not reach statistical significance. The semi-quantitative nature of the Western blot method, as well as the variability in how cells are hit by alpha radiation, are likely contributors to the difficulties in reaching significance for these relatively low-magnitude changes.

### 3.5 Expression of pro-and anti-inflammatory markers in irradiated HMC3 cells

To evaluate the effect of radiation on downstream cytokine release in HMC3 cells, mRNA expression of pro-and anti-inflammatory cytokine genes was examined. An increased expression of IL-1β, a key pro-inflammatory cytokine, was observed 6 h post-irradiation, across all radiation types. There was a clear trend of elevated IL-1β expression with a greater proportion of high-LET radiation, as depicted in Figure 5A. After 2 Gy irradiation with mixed beam and α-particles, IL-1β expression was significantly higher than after exposure to X-rays alone. This trend continued 24 h later; however, statistical significance in IL-1β expression for α-particles alone was not reached (Figure 5A).

The fractionated irradiation scheme, illustrated in Figure 5B, revealed a strong induction of IL-1β expression across all radiation modalities 24 h post-irradiation. However, there was a clear trend of IL-1β expression returning to baseline levels 10 days later, and completely reverting to baseline within 2 weeks after the last irradiation. ELISA results showed an increased IL-1β secretion, particularly with high-LET radiation (Figure 5C). Notably, cells exposed to 2.0 Gy of α-particles exhibited significantly higher secretion compared to X-ray irradiated cells, supporting the correlation between radiation type and IL-1β secretion. The intracellular protein levels of IL-1β assayed with flow cytometry were also consistent with our mRNA and ELISA findings and showed an increase at 6 h, which returned to the basal level at 10 days (Figure 5D). TNF-α, another well-known pro-inflammatory cytokine, showed the same pattern with a slightly higher magnitude compared to the IL-1β (Figure 5E).

We also analyzed the mRNA expression of IL-12α and IL-18 genes post-irradiation. At 6 h after acute exposure, only minor changes were observed (Figure 5F), so the 24-h analysis was omitted. With the fractionated protocol (Figures 5G,H), a moderate increase in transcription of both cytokine genes was observed 24 h after the last fraction across all modalities. However, 10 days post-irradiation, transcription significantly decreased, exhibiting a V-shaped pattern, and reaching basal levels within 2 weeks. Subsequently, we showed that phagocytic capacity of HMC3 cells following fractionated treatment displayed a statistically significant increase after α- and mixed beam compared to X-ray irradiation (Figure 5I).

Next, we examined the expression of IL-10, a key anti-inflammatory cytokine (Figure 6A). Minimal differences were observed in IL-10 mRNA levels at both six-and 24-h post-irradiation across all modalities, except for cells treated with 2.0 Gy of α-radiation, which showed a two-fold reduction in IL-10 expression at 6 h. With the fractionated protocol, a slight decrease in IL-10 expression was observed 24 h after the last fraction, but RNA levels returned to baseline 10 days post-irradiation (Figure 6B). The intracellular level of IL-10 protein showed an increase compared to the unirradiated group at an early time point, i.e., 6 h, and returned to basal level 10 days after the last irradiation (Figure 6C).

Furthermore, gene expression analysis of CD163 and CD206 as anti-inflammatory markers at 10 days and 2 weeks post-fractionation showed no significant shift toward anti-inflammatory phenotype among the groups (Figure 6D). Consistent with this result presence of surface marker CD206 and cell size 10 days post fractionated irradiation showed a similar level among all the groups, as depicted in Figures 6E,F. Figure 6G is summarizing the DNA damage and inflammatory response up to two weeks after fractionated high-LET radiation exposure.

## 4 Discussion

Both low-and high-dose radiation therapy has been used to address conditions such as amyloidosis and more recently AD (Hall et al., 2022; Jebelli et al., 2022; Kaul et al., 2023). The use of NCT to target AD has been suggested as a potential treatment approach that allows utilization of both high-and low-LET radiation to eliminate amyloid plaques. However, in the case of AD where the targets of irradiation are extracellular molecules, aggregated amyloid peptide, the proposed procedure may affect the surrounding brain cells including neural and glial cells (Fukuda, 2021; Jin et al., 2022). In the current study, we investigated the inflammatory and toxicity responses of microglial cells to high-and low-LET radiation, as two components of the NECTAR-investigated alternative treatment for AD, alone and in combination, in the context of single-and multi-fraction irradiation schemes.

*γ*H2AX foci formation assay showed that X-rays and mixed beam (75% X-ray) delivered via acute scheme induced more foci and, thus, more DSBs 30 min after treatment, than α-irradiation. However, the decrease in number of γH2AX foci from 30 min to 24 h was much faster for X-rays than for α-irradiation. This argues for more clustered damage in cells receiving a higher proportion of high-LET radiation. Consistent with γH2AX results, mRNA levels of DNA damage response genes exhibited a more pronounced increase at the later time point (24 h) following high-LET and mixed beam (25 or 50% low-LET) irradiation compared to low-LET one. The inhibitory activity of one of those genes, *CDKN1A*, on cell cycle progression allows cells to pause in the G1 phase, enabling recovery from irradiation damage (Bartek and Lukas, 2001) and high-LET α-particles induce a more pronounced increase in expression of *CDKN1A* in comparison to an equivalent dose of low-LET radiation (Fournier et al., 2004). Corroborating our findings on gradual increases in mRNA levels of DNA damage response genes, Azzam et al. reported comparable increase in expression of *CDKN1A* in α- and γ-irradiated human fibroblasts at an early time point. However, these enhanced levels were attenuated in γ-but not in α-particle irradiated cells at later time points (Azzam et al., 2000).

Low-LET radiation in principle distributes energy extensively across the target volume (Mladenova et al., 2022) and approximately 56% of DNA damages are attributed to *de novo* generated reactive oxygen species (ROS) rather than directly from the low-LET radiation (Falk et al., 2010). Therefore, euchromatic regions, due to their more open structure, are more susceptible to such lesions. On the other hand, high-LET irradiation predominantly focuses energy along the trajectories of the particles, leading to clustered DNA damages of various types in both euchromatic and heterochromatic regions (Asaithamby and Chen, 2011). Heterochromatin is less susceptible to DNA damage because of its compaction; however, when damage do occur, local chromatin decondensation and DNA repair require more time (Cann and Dellaire, 2011). This is evidenced from transmission electron microscopy studies, which demonstrate a progressive increase in the size and quantity of clusters of DSBs for up to 5 h following high-LET irradiation, which interfere with efficient DNA repair (Lorat et al., 2016).

The outcome of the activation of the DNA damage response after a single exposure to either high-LET, low-LET, or mixed beam, depends on the efficiency of the repair system and the complexity of the DNA lesions. Indeed, exposed cells can either repair the damage or undergo cell death or cellular senescence (Adjemian et al., 2020). Our findings showed comparable genotoxicity for high and low-LET treatments, albeit slightly leaning toward greater toxicity from high-LET after acute exposure. Although the slopes of survival curves for α-particles exhibited a linear and X-rays displayed a linear quadratic response, the similarity in response between X-rays and α-particles was unexpected since RBE values in the range of 3–8 were reported for many human cell culture models previously (Sgouros et al., 2010). Our data from HMC3 cells instead suggests RBE of α-radiation for approximately 20% survival being around one which indicates that high-LET α-particle radiation is not more damaging than low-LET X-rays for HMC3 cells in our experimental context. This suggests that HMC3 cells have efficient and robust DNA repair mechanisms that can effectively repair DNA damages from both high-LET and low-LET radiation which can diminish the expected differences in toxicity. The embryonic origin of HMC3 may contribute to this capability (Dello Russo et al., 2018). The inherent pluripotency properties of embryonic cells are related to a more euchromatic structure, which may facilitate repair induced by high-LET radiation (Ugarte et al., 2015; Svetlicic et al., 2020). Despite the detection of more DSBs 24 h post acute-irradiation in α-versus X-ray exposed cells, the overall cellular response over an extended time period did not translate into higher toxicity. Interestingly, exposure to a total of 2 Gy of mixed beam (50% of each α- and X-ray) also resulted in a comparable cell toxicity in comparison to an equivalent dose of each radiation alone which further confirms the relatively similar long-term response for both high-LET and low-LET radiation in HMC3 cells at this dose range.

Fractionated compared to acute exposure to the same total dose and radiation quality did not significantly alter the cellular response in terms of the average number of DSBs per cell assayed 24 h after (the last) exposure; however, there was a significant downward trend after low LET-and mixed beam radiation at the median value of γH2AX foci. The response at the mRNA level for DNA damage response genes (averaging around 2-fold increase) as well as the cell viability instead exhibited a consistent pattern between cells subjected to acute or fractionated low LET radiation. This is interesting since the X-ray fractionated radiotherapy is based on a faster repair of normal versus cancer cells before the next dose hits tumor. In our setting such trend was only seen in clonogenic survival experiments. Nevertheless, in a murine study, accumulation of unrepaired DSBs was observed during fractionation in all tested tissues after 5 fractions of 2 Gy whole-body gamma irradiation (Rübe et al., 2010). Additionally, a separate study involving fractionated low-dose exposure (0.05 Gy × 10) in mouse thymus also showed an accumulation of DNA lesions, and similar foci numbers as after the 0.5 Gy single exposure (Pogribny et al., 2005). The lack of a strong sparing effect after fractionated low-LET in this study could potentially be due to the relatively high dose used for this cell type, as demonstrated by the presence of residual foci at 24 h time point.

Notably, the response at the level of DNA damage response genes (*CDKN1A* and *MDM2*), cell viability and survival, was higher after fractionated compared to acute mixed-or high LET irradiation. Interestingly, acute exposure to a mixed beam consistently showed a higher DNA damage response activation compared to high-LET radiation alone; however, fractionation caused a slightly higher response to high-LET compared to mixed beam treatment. Our findings suggest a notable difference in the effect of fractionation on HMC3 cells, depending on the LET, where fractionation of low-LET radiation improves the cell survival compared to single dose administration whereas high-LET radiation fractionation has a stronger toxic effect than single dose treatment. We suspect that HMC3 cells fail to repair DNA damages adequately before the subsequent fraction, due to the more clustered nature of high-LET-induced lesions. Therefore, several doses lead to a cumulative effect of damages which may overwhelm the repair mechanisms in HMC3 cells, while in case of acute exposure, cells are able to more effectively repair the lesions in a LET-independent manner.

DSBs are able to activate the inflammatory pathway through the cGAS-STING-NF-κB signaling pathway in cases of very high doses of low LET radiation, via micronucleus (MN) formation and their subsequent rupture in the cytosol (Mackenzie et al., 2017). The presence of MN after fractionated high LET was also noted in images taken for the γH2AX analysis (Supplementary Figure S2). Few studies examined the induction of cGAS/STING in response to high LET radiation, it appears likely that broken DNA is present to a higher extent due to the higher proportion of DSBs, complex damage, deletions, and MN (Harding et al., 2017). Although leakage of DNA fragments to the cytosol also may occur, the full innate immune activation was shown to be dependent on progression into mitosis (Mackenzie et al., 2017), suggesting MN formed from lagging acentric chromosomes as the main contributors. Our results on activation of these pathways at early time points after the fractionated scheme showed that all radiation qualities are able to activate the phosphorylation of STING, which in turn activates different other pathways including the NF-κB pathway (Mackenzie et al., 2017). We observed an earlier activation of STING, with a higher magnitude, compared to NF-κB pathway activation. The level of activation remained high up to 6 h after fractionated exposure. NF-κB can be also activated through alternative pathways such as ROS production, which serves as the primary source of DNA damage during low-LET irradiation. Scavenging ROS by antioxidants demonstrated a significant inhibitory effect on the induction of NF-κB (Janssens and Tschopp, 2006). Another alternative pathway for NF-κB activation is through the phosphorylation of ATM which occurs after the formation of radiation-induced DNA lesions (Zhao et al., 2020). Our observation on cGAS-STING pathways also showed the activation of STING in irradiated cells at an earlier time point, indicating a pulsatile activation of this protein. This pattern is likely extended to other proteins associated with DSBs, such as ATM and p53, following each radiation fraction.

Our finding on downstream cytokine release revealed that exposure to high-LET radiation and a mixed beam (25% X-ray) induced a stronger pro-inflammatory response compared to low-LET radiation, particularly when using a fractionated scheme, as evident from both increased mRNA expression and protein secretion levels of IL-1β. This finding was further corroborated by intracellular IL-1β and TNF-α levels at earlier time points, although the magnitude was not as high as observed at the mRNA level. A faster response is expected at the mRNA level since the time point used to detect intracellular proteins was earlier than that for the assessment of secreted protein. Therefore, high-LET radiation has the capability to induce a shift in cells toward the pro-inflammatory and activated phenotype, promoting a pro-inflammatory state and enhancing their phagocytic capacity. This finding was corroborated by other studies and our own data, which have shown that the production of pro-inflammatory cytokines increase the phagocytic capacity of cells (Mandell, 1995). Interestingly, the irradiated cells not only reverted to their basal inflammation state at the later time point, but the expression of pro-inflammatory cytokines such as IL-12 and IL-18 also decreased compared to non-irradiated cells, thus, showing their anti-inflammatory state. Consistent with previous findings, a lower IL-10 mRNA level at the early time point was observed, which then reverted to basal level. However, our results on anti-inflammatory markers such as CD163 and CD206 showed no significant alteration at this late time point.

Mechanistically, it is important to be aware that high LET irradiation will induce a strong inflammatory response when given daily, which is relevant to consider from the perspective of targeted alpha therapy and carbon ions as well. From the therapeutic point of view and also based on how NCT is given currently, a single exposure or longer intervals between high LET fractions would likely be preferred from a safety perspective to allow a more balanced and well-tolerated response both in relation to inflammation and cell survival. Optimally, direct fragmentation of amyloid plaques by irradiation, together with increased phagocytic clearance, could potentially compensate for the loss of microglial cells.

While this study provides valuable insights into the effect of BNCT on microglial cells and provide ground for further exploration, a few limitations should be considered. Although the HMC3 cell line is a well-characterized and well-known model in the field of neuroinflammation with numerous publications supporting its use, there is a possibility that this model may respond differently to radiation exposure compared to primary microglial cells, especially outside of more complex contexts involving other brain cell types. Further studies would be needed to discriminate if the reduced survival mainly is caused by a reduction in microglial cell proliferation or an increase in microglial cell death. The similarities between clonogenic survival and cell viability data however suggest that cell death is the main pathway, and colonies were not strikingly smaller while their numbers differed. To enhance the translational relevance of our work in relation to neurodegenerative diseases, it would be valuable with disease-specific markers such as fluorescent amyloid or α-synuclein fibrils for the phagocytosis readout. They do however produce a higher level of variability compared to the well-characterized and reproducible commercial zymosan uptake kit that was chosen here. Zymosan particles specifically target microglial phagocytic receptors, providing a precise measure of basic phagocytic activity. Moreover, additional biochemical and morphological readouts of microglial activation would aid in proving that phenotype. To counteract the known high baseline reactivity for cultured microglia, data was always normalized to the non-irradiated group to minimize any background effects. We also believe that the strong difference after fractionation versus acute exposure for high LET pinpoints that the cells are not merely strongly responsive to any type of treatment, which show that also simple cell models have advantages.

In summary, our study aimed at investigating of responses to different radiation qualities and irradiation schemes in microglial cells. High-LET radiation induced more protracted DNA damage and activated DNA damage response genes compared to low-LET. Fractionation affected cellular response differently based on LET, with high-LET radiation showing higher toxicity and low-LET, in turn, tended to improve cell survival. We have shown that radiation-induced inflammation occurred via cGAS/STING/NF-κB pathway activation with the pro-inflammatory response enhancing the phagocytic capacity of the microglial cells. Importantly, all alterations returned to basal levels at 2 weeks after exposure. Several studies proposed that neuroinflammation may have neuroprotective function in the early stages of AD by controlling amyloid accumulation. However, as the disease progresses, these processes could transition to exerting harmful effects on neurons (Gratuze et al., 2018). Despite limitations in the current setting such as using an immortalized cell line, our experiments allowed us to evaluate the eventual toxicity and activated pathways after irradiation using a mixed beam mimicking the complex BNCT beam in a microglial cell model, which provides a starting point for further studies on primary rodent microglial cells and animals. Our findings offer valuable insights into optimizing possible high-LET radiation therapy for neurodegenerative diseases such as AD.

## Data Availability

The original contributions presented in the study are included in the article/Supplementary material, further inquiries can be directed to the corresponding author.
