# Supplementary material for: Fractionated alpha and mixed beam radiation promote stronger pro-inflammatory effects compared to acute exposure and trigger phagocytosis
Source: Front Cell Neurosci. 2024 Dec 9;18:1440559. doi: 10.3389/fncel.2024.1440559 (PMC11663654; doi:10.3389/fncel.2024.1440559)
Supplement: Supplementary file 1 [file Image_1.pdf]

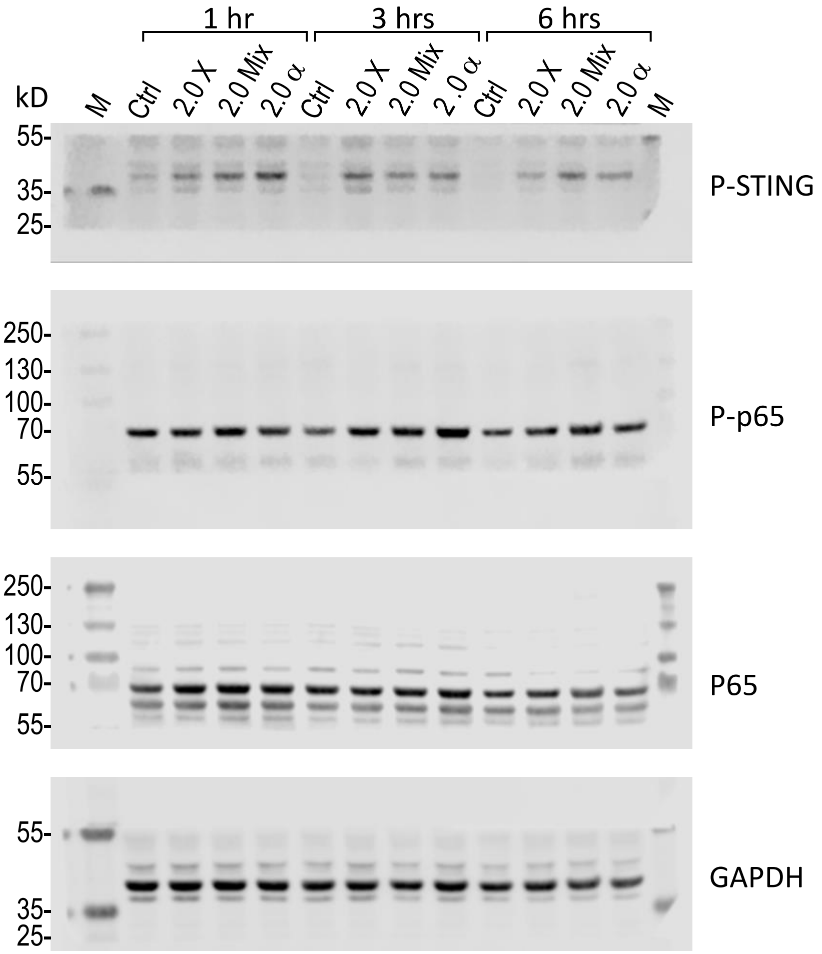


**Supplementary Fig. 1.** Proteins were separated using 4-12% Bis-Tris gradient gels in 1xMES running buffer (Invitrogen™, USA) and then transferred to a nitrocellulose membrane (Thermo Scientific, USA). Membranes were cut horizontally into three strips to analyze proteins >55 kD (to assay with p65 and P-p65 antibodies); 25-55 kDa (P-STING and GAPDH antibodies); <25 kD (histone H3 antibody, used in some experiments as additional loading control, not shown).
