# Supplementary material for: Fractionated alpha and mixed beam radiation promote stronger pro-inflammatory effects compared to acute exposure and trigger phagocytosis
Source: Front Cell Neurosci. 2024 Dec 9;18:1440559. doi: 10.3389/fncel.2024.1440559 (PMC11663654; doi:10.3389/fncel.2024.1440559)
Supplement: Supplementary file 2 [file Image_2.pdf]

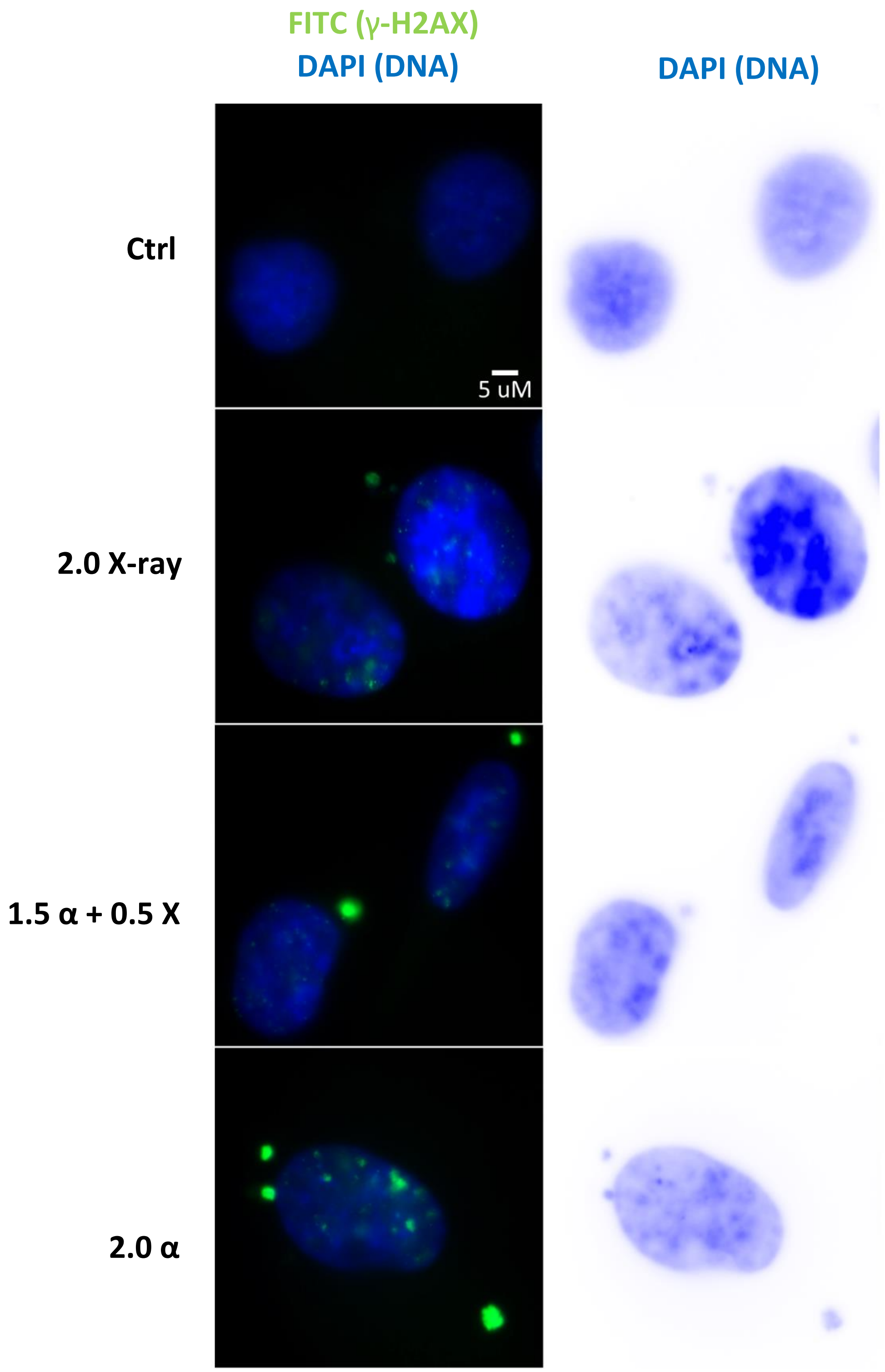

Supplementary Fig. 2: Representative fluorescence microscopy images of micronuclei (green/blue bodies around the nucleus) at 24 h after the last radiation fraction in the different groups receiving a total of 2 Gy X-rays, 1.5 Gy α-particles plus 0.5 Gy X-rays, 2 Gy α-particles, as well as control (Ctrl). Left panel: γ-H2AX staining using a FITC-labelled secondary antibody (green) together with DNA staining by DAPI (blue) displaying nuclei as well as micronuclei. Right panel: DNA staining displaying nuclei as well as micronuclei stained with DAPI (blue). Images were captured using a fluorescent microscope using a 100× objective.
